# A gene browser of colorectal cancer with literature evidence and pre-computed regulatory information to identify key tumor suppressors and oncogenes

**DOI:** 10.1038/srep30624

**Published:** 2016-08-01

**Authors:** Min Zhao, Yining Liu, Fuda Huang, Hong Qu

**Affiliations:** 1 School of Engineering, Faculty of Science, Health, Education and Engineering, University of the Sunshine Coast, Maroochydore DC, Queensland 4558, Australia; 2 Center for Bioinformatics, State Key Laboratory of Protein and Plant Gene Research, College of Life Sciences, Peking University, Beijing 100871, P.R. China

## Abstract

Colorectal cancer (CRC) is a cancer of growing incidence that associates with a high mortality rate worldwide. There is a poor understanding of the heterogeneity of CRC with regard to causative genetic mutations and gene regulatory mechanisms. Previous studies have identified several susceptibility genes in small-scale experiments. However, the information has not been comprehensively and systematically compiled and interpreted. In this study, we constructed the gbCRC, the first literature-based gene resource for investigating CRC-related human genes. The features of our database include: (i) manual curation of experimentally-verified genes reported in the literature; (ii) comprehensive integration of five reliable data sources; and (iii) pre-computed regulatory patterns involving transcription factors, microRNAs and long non-coding RNAs. In total, 2067 genes associating with 2819 PubMed abstracts were compiled. Comprehensive functional annotations associated with all the genes, including gene expression profiles, homologous genes in other model species, protein-protein interactions, somatic mutations, and potential methylation sites. These comprehensive annotations and this pre-computed regulatory information highlighted the importance of the gbCRC with regard to the unexplored regulatory network of CRC. This information is available in a plain text format that is free to download.

Colorectal cancer (CRC) is the third most diagnosed cancer, resulting in high mortality rates worldwide^1^. In 2012, approximately 1.4 million individuals were diagnosed with CRC across the globe. The highest rates of incidence are in developed countries such as New Zealand, Australia, and Western Europe. Not surprisingly, the prognosis often decreases substantially for those individuals in which the disease has metastasized to other tissues and organs. Only 11% (colon cancer) and 12% (rectal cancer) of patients with distant metastases survive the first five years^2^. Therefore, the development of effective biomarkers to monitor CRC development is needed to improve the diagnosis rate at late stages.

Similar to other malignancies, CRC is caused by uncontrolled cell growth. However, it is remarkably heterogeneous at multiple cellular and molecular levels. For example, uncontrolled cell proliferation in CRC may result from the abnormal gene expression of tumor suppressors and oncogenes^3^, promoter methylation^4^, copy number alterations of cancer genes that control cell proliferation and death^5^, microRNA mutations and abnormal expression^6^, and long non-coding RNAs^7^. Thus, it is critical to prioritize the genetic and genomic suspects with an understanding of their tumorigenic mechanisms in future studies.

Before the development of high-throughput genome-wide sequencing, countless biochemical experimental and genetic association studies resulted in the accumulation of much data without systematic integration. To reuse and mine these published articles, we developed the first evidence-based gene resource for CRC by extensive literature curation and data integration at http://gbCRC.bioinfo-minzhao.org/. By extensive literature curation and data integration, it is expected that our gbCRC will be valuable for researchers to efficiently obtain functional and mutational data on CRC genes.

## Results

### Gene collection pipeline

**Figure 1:**
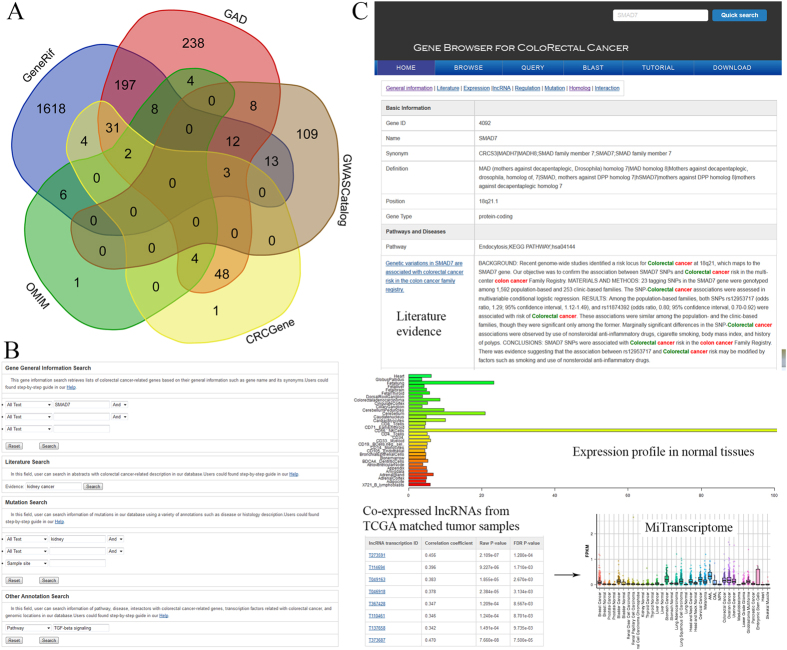
Web interface of the gbCRC. (**A**) The shared CRC-implicated genes across multiple data sources. (**B**) The web interface of the gbCRC, including the basic information, curated literature, gene expression and pre-computed lncRNA co-expression results using TCGA CRC tumor samples. (**C**) The overlapping of 21 CRC-implicated genes with tumor suppressor genes and oncogenes from TSGene 2.0.

To address the genetic complexity of CRC, we conducted extensive data integration and literature curation. The final non-redundant 2309 relevant genes were stored in a publicly available database of human CRC-implicated genes. The CRC gene collection was mainly based on five data sources (Fig. 1A), including OMIM (Online Mendelian Inheritance in Man)^8^, GAD (The Genetic Association database)^9^, genes manually curated from GeneRif^10^, genome-wide association studies from GWASCatalog^11^ and meta-analyses results of genetic association studies from the CRCGene database (http://www.cphs.mvm.ed.ac.uk/projects/CRCgene/index.php). As the most authoritative compendium of human disease-related genes, OMIM did not include many genes. We obtained 25 genes associated with CRC from OMIM. The GAD database is an archive of published human genetic association studies, which contains curated information on each candidate gene. In total, we collected 279 unique human genes from GAD from 637 published studies. Additionally, 11 candidate genes were downloaded from three genome-wide association studies in the GWASCatalog database. From the CRCGene database, 95 unique human genes were collected. After combining data from these four publically-available genetic resources, we created a non-redundant gene list with 691 human genes.

To provide a detailed and precise regeneration gene resource with supporting literature evidence, we performed an extensive literature query of the GeneRif database on January 10, 2015 using the Perl regular expression to match those sentences with both “colorectal” and “cancer” keywords: [(colorectal OR colon OR rectal OR bowel) AND (cancer OR tumor OR carcinoma OR adenocarcinoma)]. In total, we retrieved 7581 relevant short descriptions related to CRC from 5378 PubMed abstracts. GeneRif (Gene Reference Into Function) is a collection of short descriptions on the function of different genes in the Entrez Gene database^10^. However, these short descriptions from GeneRif did not contain full abstracts for accurate data curation. Therefore, we extracted the 5378 abstracts with full text for manual checking. In general, literature curation had three steps: (i) grouping the 5378 extracted abstracts based on their computed similarity scores using the ELink function in Entrez; (ii) collecting contents related to CRC from grouped abstracts; and (iii) manually collecting gene names from the CRC-related sentences and mapping the gene names to Entrez gene IDs. These three curation steps provided high-quality data by cross-checking if and how the curated abstract was related to CRC. For a unified functional annotation, we regarded Entrez gene IDs as the unique key in all the data tables of our gbCRC database to link the same genes from different bioinformatics resources. To obtain accurate literature evidence, we collected the species information and the gene alias and manually mapped this information to the official HUGO gene symbol. For example, two negative results were described in the following sentences: “*study does not support a role of COX2 and UGT1A6 genetic variations in the development of colon cancer*”^12^ and “*neither genetic variation nor allele-specific expression at TGFBR1 is likely to be a major colorectal cancer risk factor*”^13^. In addition, in the first sentence, the gene name *COX2* was one of the synonyms of *PTGS2* in the current Entrez gene database. Thus, we deleted the associated relationship from the literature with the *PTGS2* gene. As some of the studies were not conducted in the human, we mapped the collected genes to their corresponding human homologous groups using the NCBI HomoloGene database as described in previous studies^14^,^15^,^16^. In total, we pinpointed 1618 Entrez human homologous genes from 5515 PubMed abstracts. By integrating the 691 genes from other public databases, we consolidated 2309 human genes in Table S1.

All curated genes and relevant bioinformatics annotations were stored in a MySQL-based database on a Linux server. In the gbCRC, we provided two approaches to access our data: text-based query (Fig. 1B) and sequence similarity-based BLAST search. Users can retrieve a list of genes with their interesting annotations by using the text-based query. Users can also annotate unknown sequences via BLAST against all the DNA and protein sequences in the gbCRC. Lastly, users can search the data in a variety of ways, including the number of supporting references, the highlighted KEGG pathway, and genomic positions.

The annotations of a representative gbCRC gene were classified into eight categories by clicking the labels “expression”, “general information”, “homolog”, “literature”, “interaction”, “lncRNA”, “mutation”, and “regulation” (Fig. 1C). The keyword-marked references were provided on the “literature” page. The pre-computed co-expression correlation coefficients and the corrected statistical *P*-values between CRC-implicated genes and lncRNAs in matched cancer samples were presented on the “lncRNA” page. The “homolog” page provided the homologous genes from another 11 model species such as the mouse. On the “regulation” page layout, which listed the interactions with transcription factors, there was information on post-translational modifications and potential methylation sites.

### Potential role of retinoic acid in colorectal cancer

**Figure 2:**
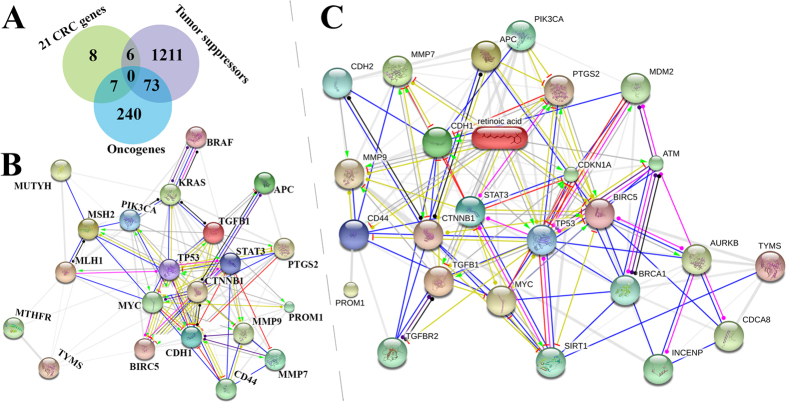
The role of retinoic acid in colorectal cancer. (**A**) The tumor suppressors and oncogenes in the gbCRC. (**B**) The interaction of 21 well-studied CRC-related genes using STRING. (**C**) The interaction of the 21 genes in (**B**) with retinoic acid using STITCH.

To provide an overview of the data quality, we counted the number of supporting references for the collected 2,309 CRC-implicated genes. This preliminary statistical analysis not only highlighted the high tumor susceptibility of these well-studied genes, but also confirmed the heterogeneous genetic events of CRC. In total, there were 21 genes with more than 30 concordant positive supporting references. The majority of the genes (2172 genes, 94.06% of the total number of genes) had less than ten supporting references. These 21 genes were further classified according to their roles in cancer. We found six tumor suppressors and seven oncogenes (Fig. 2A). The remaining eight genes may also have contributed to cancer progression, but their roles in its suppression or progression were not immediately clear. Further functional enrichment analysis revealed that 17 of the 21 genes increased with tumor incidence and 15 of them regulated cell apoptosis. More importantly, these tumor suppressors and oncogenes were highly connected to each other in CRC (Fig. 2B).

A fundamental goal of biomedical research is to discover effective drugs to cure human diseases. To demonstrate the use of our gbCRC, we constructed a compound-gene interaction network using the STITCH database (http://stitch.embl.de/). Intriguingly, the compound-gene interaction enrichment module revealed that 16 of the 21 well-studied CRC-implicated genes were predicted to interact with retinoic acid (C_20_H_28_O_2_) (Fig. 2C). As the most active metabolite of vitamin A, retinoic acid can trigger programmed cell death in certain types of tumor cells^17^. Additionally, aberrant retinoic acid metabolism is implicated in tumorigenic events in cancer cell lines^17^. Furthermore, three metabolic enzymes for retinoic acid, CYP26A1, CYP26B1 and LRAT, are significantly overexpressed in CRC^18^. These enzymes were also significantly associated with poor prognosis in colorectal cohorts^18^. Our rudimentary analysis of a potential gene-compound interaction network allowed us to relate retinoic acid to CRC. Retinoic acid mediates the functions of vitamin A for individual growth and development, and retinoids can interact with estrogen signaling in breast cancer^19^. Although retinoid signaling is often compromised early in cancer progression^19^, its broader effect on CRC has been recognized recently^20^. Since a reduction in retinoid signaling may be required for cancer progression, retinoids can be used to induce differentiation and arrest proliferation in a clinical setting. Although it is ideal to treat cancer with retinoids, the delivery of retinoids to patients is hindered by their rapid metabolism. Regardless, our constructed gene-compound network may benefit cancer prevention strategies related to retinoids for CRC patients. Taken together, these results offer insight into the functional importance of several well-studied CRC-related genes with ample supporting evidence for their relevance to retinoid signaling. More extensive metabolic network analysis may provide more insight into these important compound in cancer progression^21^,^22^,^23^.

### Abundant mutations within well-studied protein-coding tumor suppressors and oncogenes

**Figure 3:**
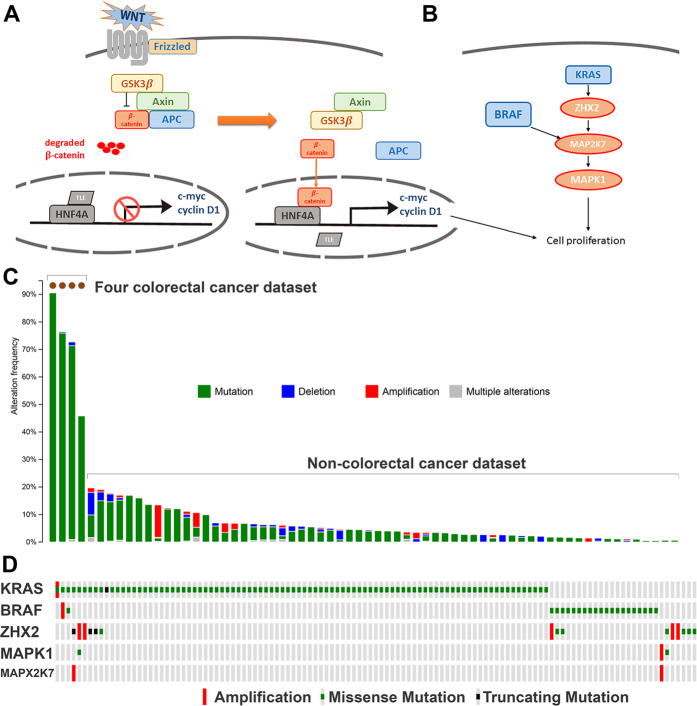
Mutational frequency of two key biological pathways in colorectal cancer. (**A**) The somatic mutations of different genes from the Wnt signaling pathway in CRC. (**B**) The somatic mutations of different genes from the KRAS-BRAF pathway in CRC. (**C**) The mutational frequency of *APC* across multiple cancers. (**D**) The mutational frequency of different genes from the KRAS-BRAF pathway in CRC.

A fundamental problem in cancer biology is discovering potential tumor suppressors and oncogenes, which may help us to understand the key genetic drivers for cancer progression. As shown in Figure S1, 13 well-studied tumor suppressors and oncogenes were highly mutated in CRC. Among 212 TCGA tumor samples, 206 of the individuals had at least one mutational event (mutational rate, 97%). However, the most abundantly mutated six genes covered 205 samples: *APC* (mutational rate, 76%), *TP53* (52%), *KRAS* (42%), *PIK3CA* (20%), *BRAF* (10%) and *CTNNB1* (5%). Notably, the majority of the mutations were enriched in a few important oncogenic pathways (Fig. 3A,B). A previous study showed that *APC* is truncated in the “mutation cluster region” close to codon 1,300. The mutations often result in the loss of C-terminal APC functions. The *APC* gene, located at 5q21-q22, encodes a critical Wnt signaling regulator (Fig. 3A)^24^. In the absence of the Wnt signal, *APC* can form a complex with *AXIN1* and *GSK3B* to mediate degradation of β-catenin (*CTNNB1*) via phosphorylation. In this way, the abundance of β-catenin in cells is strictly controlled. Upon activation of Wnt signaling, however, the multi-protein complex (*APC*, *AXIN1* and *GSK3B*) is disassembled, resulting in a high concentration of β-catenin. Upon translocation to the nucleus, β-catenin triggers *HNF4A* (hepatocyte nuclear factor 4) activity by displacing the suppressive effect of TLE (transduction enhancer-like protein). This triggers the expression of important genes associated with cell proliferation (*MYC* and *CD1*)^24^. Therefore, the truncated APC can induce higher levels of β-catenin (*CTNNB1*) and its downstream cell proliferation genes. In total, these two genes accounted for 165 samples (combined mutational rate, 77.83%). Additional analysis of other genes in the Wnt signaling pathway found that HNF4A had a mutational rate of 14% (Figure S2). Both *GSK3B* and *AXIN1* had a mutational rate of 2%. For the 17 genes from the Wnt family, 14 were sporadically mutated with a rate less than 1%. The remaining three (*WNT3*, *WNT5A*, *WNT10B*) had a mutational rate of 2%. For the ten genes from the Wnt signaling receptor family (FZD genes), FZD3 had the highest mutational rate of 6%. Combining all the genes, 186 samples had at least one mutational event, accounting for 88% of the TCGA cohort. Mutations within *TP53* are expected in CRC. By comparing the pan-cancer mutational pattern of *APC* and *TP53*, we found that the high mutational rate of *APC* is specific for CRC, placing it at the top of mutated cancer cohorts (Fig. 3C). On the contrary, *TP53* is highly mutated across multiple cancers, not just in CRC (Figure S3). This specificity of the APC mutation may indicate that it is a top driver in CRC initiation. *TP53* is more important at the late stage of cancer development.

The other most mutated oncogenes KRAS and BRAF belong to the RAS signaling pathway (Fig. 3B). Their downstream molecules include ZHX2, MAPK2K7 and MAPK1. In total, these five genes had a combinational mutation rate of 55% (Fig. 3D). Despite MAPK2K7 and MAPK1 having a mutational rate of only 1%, the direct downstream gene of KRAS (ZHXS) was mutated in 15 individuals.

### Role of microRNAs in colorectal cancer

**Figure 4:**
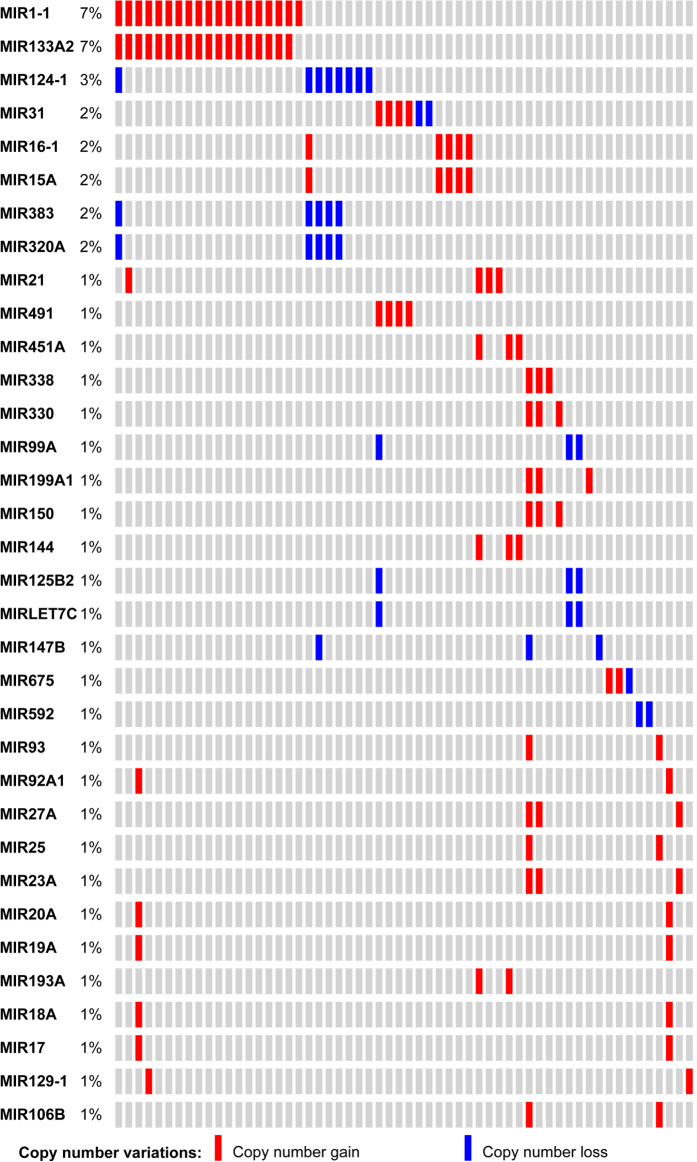
Mutational landscape of the 34 miRNAs with supporting references.

In recent years, an increasing number of miRNAs has been associated with CRC^6^. We collected 119 miRNAs with important roles in CRC. To assess their potential functions, we first used the TCGA mutational data to isolate those miRNAs with mutations in tumor samples. Because miRNAs are relatively short in terms of nucleotide sequence, single nucleotide variants (SNVs) may not be easily detected. Therefore, we investigated the meditate scale copy number variations (CNVs) for the miRNAs. In total, we found 34 miRNAs with CNVs in the 53 CRC patients from a cohort of 212 TCGA tumor samples (Fig. 4). For the top eight miRNAs with a mutational frequency of approximately 2%, only four miRNAs gained more copy number instead of losing copy number. To further explore the functional distribution of these four miRNAs with more copy number, we performed a target-based functional enrichment analysis using DIANA-miRPath (Table 1). Not surprisingly, the majority of the enriched pathways were related to key cancer pathways such as the cell cycle and p53 signaling pathways. For the cell cycle pathway, 42 genes were the targets of the five miRNAs (Figure S4, corrected P-value = 4.48E-20). The top two mutated miRNAs MIR1-1 and MIR133A2 had a copy number gain in approximately 7% of patients. Interestingly, these two miRNAs had a tendency to co-mutate in the same patients, which may be explained by their relative proximity to each other within the genome. MIR1-1 associates with MET-dependent proliferation in CRC^25^. However, these two miRNAs are down-regulated in CRC^26^,^27^, which conflicts with the consistent gene copy number gain. With more copy number, gene expression is often relatively high in agreement with the gene dosage effects. Further experimental validation is needed to test whether the down-regulation of these two miRNAs was caused by other epigenetic mechanisms such as DNA hypermethylation^28^. We also observed a similar scenario with two other miRNAs (*MIR15A* and *MIR16*). These two miRNAs cluster at the genomic region of 13q14.3, which is frequently deleted in chronic lymphocytic leukemia. *MIR16* has also been reported to be down-regulated in CRC^29^. Furthermore, two additional miRNAs, *MIR31* and *MIR21*, had more gene copies in the TCGA CRC cohort. Both *MIR31*^30^ and *MIR21*^31^ are up-regulated in CRC and are potential clinical biomarkers. In summary, the clinical significance of the MIR1-1/MIR133A2 and MIR15A/16 cluster in CRC has not been fully elucidated. Further tests should focus on their dysregulation with respect to their epigenetic effects.

### Copy number mutations of highly connected genes

**Figure 5:**
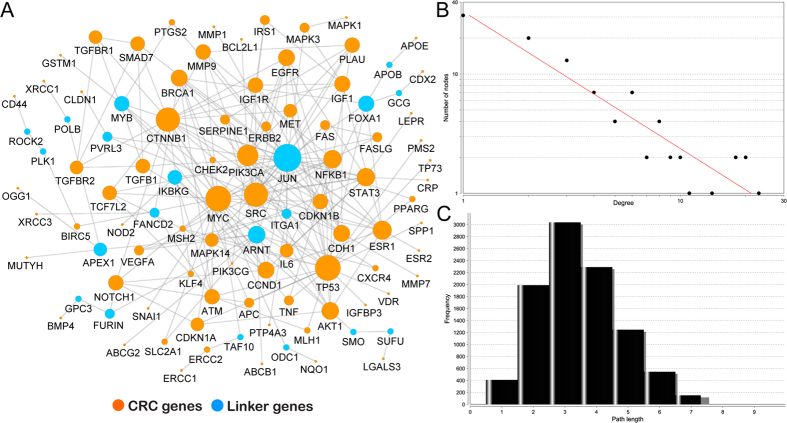
Reconstructed CRC map based on 114 genes with ten or more supporting references. (**A**) The 87 genes in orange are genes in our gbCRC. The remaining 12 genes in red are linker genes that bridge the 87 genes. (**B**) The degree distribution. (**C**) The short path length frequency.

To improve the systems level understanding of CRC, we constructed a pathway-based protein-protein interaction map for CRC. To avoid the high level of noise, we only used the 114 genes with ten or more supporting references to build the network. In addition, we only utilized reliable human PPIs summarized in a few popular biological pathways such as the KEGG and Reactome pathway database^32^ to avoid a potentially highly skewed degree distribution of physical interaction-based PPI networks. Based on our previous module searching method^33^, a sub-network from the entire human pathway-based interactome was reconstructed containing 99 genes and 204 PPIs (Fig. 5A). Of the 99 nodes, 87 were from our curated 114 CRC-related genes with ten or more supporting references. The remaining 12 were the linker genes, which formed a completely connected cellular map. Further network topological analysis also indicated that most nodes in the reconstructed map were closely connected. There were only 31 nodes with one connection, which indicated that the remaining 68 nodes had two or more connections. The degrees of all nodes followed a power law distribution *P(k) ~ k*^*−b*^, where *P(k)* was the probability that a node had connections with other *k* nodes and *b* was an exponent with an estimated value of 1.149. Previous topological analysis on the human PPI network revealed that most of nodes are sparsely connected with exponent *b* as 2.9^34^. Compared to the entire human PPI network, our network was more closely connected. This finding was also confirmed in the shortest path distribution, where approximately 55.98% of the communication between nodes was reached by only three steps (Fig. 5C).

With high modularity, the highly connected nodes may have critical roles in the transfer of cellular signals using the shortest paths. To test whether these genes are abnormally expressed or functionally relevant to CRC, we conducted a systematic examination of the genetic variants. Based on the single nucleotide variants (SNVs), copy number variations (CNVs) and abnormal expression in tumor samples, we found that 79 highly connected genes were altered in 211 of 212 CRC tumor samples. As shown in Figure S3, *APC* and *TP53*, the top two mutated genes, were mostly SNVs. Intriguingly, the other eight highly mutated genes (mutational frequency, >10%) had more copy number changes. To further explore these CNVs with potentially important roles in cancer progression, we mapped our 79 highly connected genes to the CNV data of the TCGA CRC dataset (Figure S5). The top eight CNV-based genes (*BCL2L1, MMP9, SNAI1, SRC, MYC, CDX2, PTP4A3*, and *ERBB2*) had a copy number gain, and not a copy number loss. Interestingly, these mutated genes have roles in tumorigenesis as reported in the literature. Taking *BCL2L1* as an example, it can influence tumor cell apoptosis by regulating the opening of the outer mitochondrial membrane channel. After further mapping the gene expression data of the matched tumor samples in the TCGA CRC cohort, we found that there was a concordant copy number gain and an up-regulation of *BCL2L1* (Figure S6). Our results may provide clues for the important role of *BCL2L1* in the progression of CRC by copy number gain, which induces gene expression by increasing the gene dosage effect. In summary, our reconstructed CRC map not only identifies multiple pathways related to a few known signaling pathways, but it also provides a broader mutational landscape for previously overlooked highly connected genes. Hopefully, this information will be useful for users wishing to conduct further screening on CRC patients.

## Discussion

In conclusion, we developed a literature-based knowledge base of CRC genes with comprehensive annotations. The associated comprehensive annotations and pre-computed regulatory information highlighted the importance of the gbCRC in the unexplored field of CRC development. For advanced systems biology analysis, a complete downloadable gene list with functional features is available in a plain text format. Although we performed extensive literature curation and data integration, it is important to acknowledge the difficulty of performing an error-free search. Therefore, we provided a high confidence gene list with 162 genes from 2 or more data sources and with at least 5 supporting published articles.

Our systematic analysis of CRC-related genes from the literature resulted in numerous testable hypotheses regarding critical tumor suppressors, oncogenes, miRNAs, and oncogenic pathways. Most importantly, our integrative analyses of these genes between various annotations helped to uncover several critical biological events, as well as to explain their underlying interplay in cancer progression. In addition, the massive precomputed co-expressed lncRNAs will be helpful for the CRC-specific lncRNA identification and subsequent analyses. Moreover, the curated genes are useful for the design of target-sequencing experiments on CRC. Lastly, it will be also useful for the genetic mutation prioritization and filtering for whole genome sequencing and whole exome sequencing data analysis.

To collect additional references in the future, we implemented an automatic literature search by using NCBI Entrez programming utilities, which will retrieve PubMed references based on the matching keyword(s). The Entrez reference similarity will be used to cluster the newly available references with those curated references in our gbCRC. With regard to the bioinformatics annotation, we also implemented an automatic system to integrate functional information from public data sources. The web page will be updated accordingly on an annual basis.

## Methods

### Bioinformatics annotation

To present a comprehensive functional view for each gene in the gbCRC, we provided the basic gene information, sequences and crosslinks to the NCBI Entrez gene and Homologene databases (downloaded on April 8, 2015)^35^. We also imported the BioGPS mRNA expression profiling data from both normal and tumorigenic tissues^36^. In the BioGPS database, the relative expression scores were generated from Affymetrix chips based on fluorescent intensity of different genes. For each gene, there were multiple probes on the microarray. The final intensity value for each gene was computed using the data processing algorithm GCRMA^37^. To provide a genetic mutational overview, we annotated all the genes using the mutational data from COSMIC (V72)^38^. To present a comprehensive biological pathway, we annotated the CRC-implicated genes using BioCyc^39^ and the KEGG pathway^40^. Additional regulatory information on post-translational modifications^41^, methylation sites^42^, and protein-protein interactions from Pathway Commons (V5)^32^ was also included. For advanced bioinformatics or systems biology-based analyses, we provided the downloadable gene list in a plain text format.

### Co-expressed long non-coding RNAs from TCGA matched cancer samples

To explore the co-expressed long non-coding RNAs (lncRNAs) related to the CRC-implicated genes, we downloaded the human lncRNA expression profile from Mitranscriptome^43^. The Mitranscriptome is comprised of lncRNA expression data generated from the assembly of the RNAseq data from The Cancer Genome Atlas (TCGA) tumor samples. We computed the correlation coefficients between CRC-implicated genes and the 17,250 lncRNAs in the Mitranscriptome using Spearman’s correlation based on the matched TCGA CRC samples^44^. The statistical *P*-values were calculated using R (version 2.14.0), and the false discovery rate (FDR) was adjusted for multiple testing. For each CRC-implicated gene and lncRNA pair, we set the expression correlation score to greater than 0.3 and the FDR-adjusted *P*-value to less than 0.01.

### Network construction

To present a network view for the CRC-implicated genes, we used the online tools STRING (http://string-db.org/) and STITCH (http://stitch.embl.de/) to run the network analysis on a small number of genes of interest. For the larger gene list such as the 114 curated genes with ten or more supporting references, we adopted sub-network extraction as described in a previous study^33^. To reveal the potential function of each node, topological analyses were conducted using the NetworkAnalyzer plugin in Cytoscape 2.8 (Fig. 5B,C)^45^. The degree indicated the number of connections for each node in a network^46^. The short path was used to characterize the shortest route for each node to reach another^46^. The final network visualization was performed using Cytoscape 2.8^45^. Throughout the study, the CRC-related mutational analyses were conducted using cBio portal^47^.

## Additional Information

**How to cite this article**: Zhao, M. *et al*. A gene browser of colorectal cancer with literature evidence and pre-computed regulatory information to identify key tumor suppressors and oncogenes. *Sci. Rep*. **6**, 30624; doi: 10.1038/srep30624 (2016).

## Supplementary Material

Supplementary Dataset 1

Supplementary Table 1

## Figures and Tables

**Table 1:** Significantly enriched KEGG pathways in the targeted genes of the top five microRNAs with copy number gain.

*KEGG Pathway*	*# of genes*	*# of miRNAs*	Adjusted P-values*
Cell cycle	42	5	4.48E-20
p53 signaling pathway	27	5	3.04E-16
Prion diseases	7	2	3.50E-14
Protein export	12	4	1.17E-13
Pathways in cancer	74	5	1.17E-13
Small cell lung cancer	26	5	1.32E-11
RNA transport	41	5	2.05E-11
Arrhythmogenic right ventricular cardiomyopathy (ARVC)	20	3	4.19E-11
Hepatitis B	38	5	1.89E-10
Ribosome biogenesis in eukaryotes	23	3	4.01E-09
Bladder cancer	15	5	4.01E-09
Chronic myeloid leukemia	21	5	4.65E-08
Hypertrophic cardiomyopathy (HCM)	22	3	2.24E-07
PI3K-Akt signaling pathway	65	5	2.24E-07
Spliceosome	33	3	5.28E-07
Transcriptional misregulation in cancer	40	5	7.71E-07
Ribosome	23	2	1.10E-06
Prostate cancer	23	5	1.58E-06
Legionellosis	16	5	4.20E-06
Fatty acid elongation	9	3	1.49E-05
Colorectal cancer	16	4	1.49E-05
Pancreatic cancer	20	5	1.49E-05
Protein processing in endoplasmic reticulum	35	5	0.000182503
Glioma	18	5	0.000200629
Oocyte meiosis	25	5	0.000333129
Focal adhesion	38	5	0.000352036
DNA replication	10	3	0.000580251
Lysine degradation	14	3	0.000597329
Dilated cardiomyopathy	19	3	0.00077407
Salmonella infection	18	4	0.00077407
Insulin signaling pathway	27	5	0.000819063
Pathogenic Escherichia coli infection	13	3	0.001527875
mRNA surveillance pathway	20	5	0.001527875
Shigellosis	14	4	0.001665937
Melanoma	15	5	0.002187145
Mineral absorption	11	3	0.003792775
Epstein-Barr virus infection	36	5	0.007386609
Gap junction	15	3	0.009089295
ErbB signaling pathway	16	5	0.009089295

*Adjusted *P*-values: the *P*-values of the hypergeometric test were corrected by Benjamini-Hochberg multiple testing correction.
