# Supplementary material for: A gene browser of colorectal cancer with literature evidence and pre-computed regulatory information to identify key tumor suppressors and oncogenes
Source: Sci Rep. 2016 Aug 1;6:30624. doi: 10.1038/srep30624 (PMC4967895; doi:10.1038/srep30624)

Title: A gene browser of colorectal cancer with literature evidence and pre-computed regulatory information to identify key tumor suppressors and oncogenes

Authors: Min Zhao, Yining Liu, Fuda Huang, Hong Qu

SUPPLEMENTARY DATA

Figure S1. Mutational landscape of the 13 tumor suppressors and oncogenes in the gbCRC with supporting references.

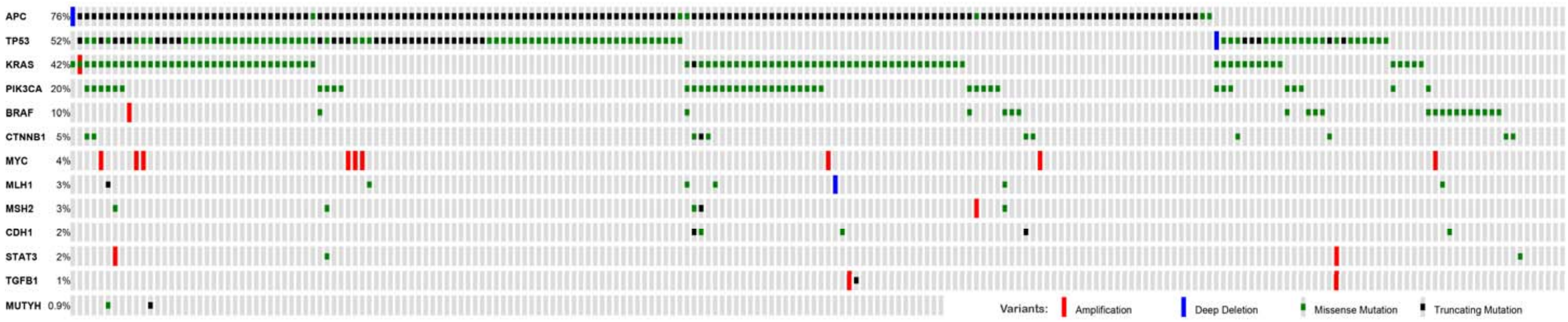

Figure S2. Mutational landscape of different genes from the Wnt pathway in the gbCRC with supporting references.

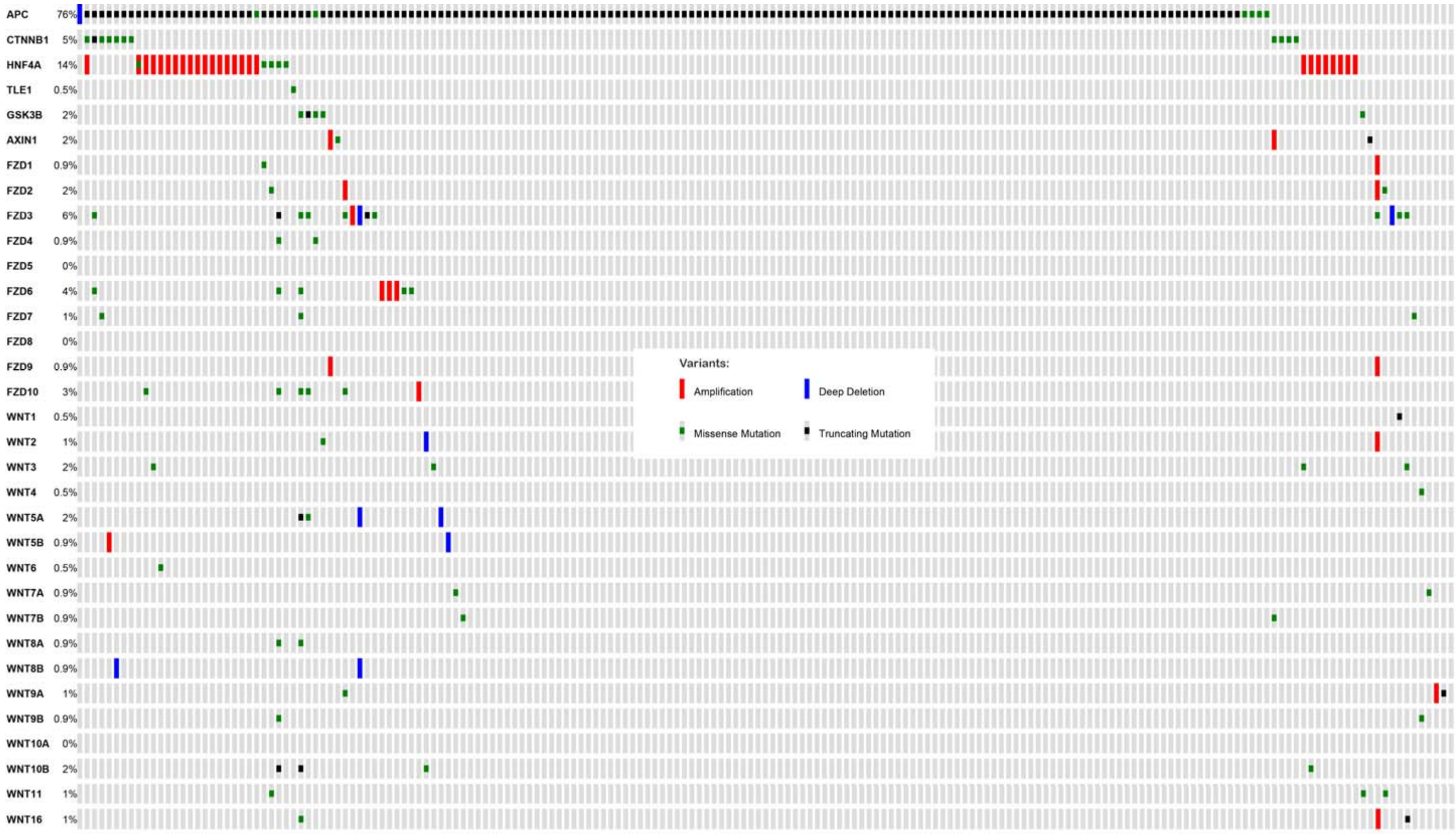

Figure S3. The mutational frequency of TP53 across multiple cancers.

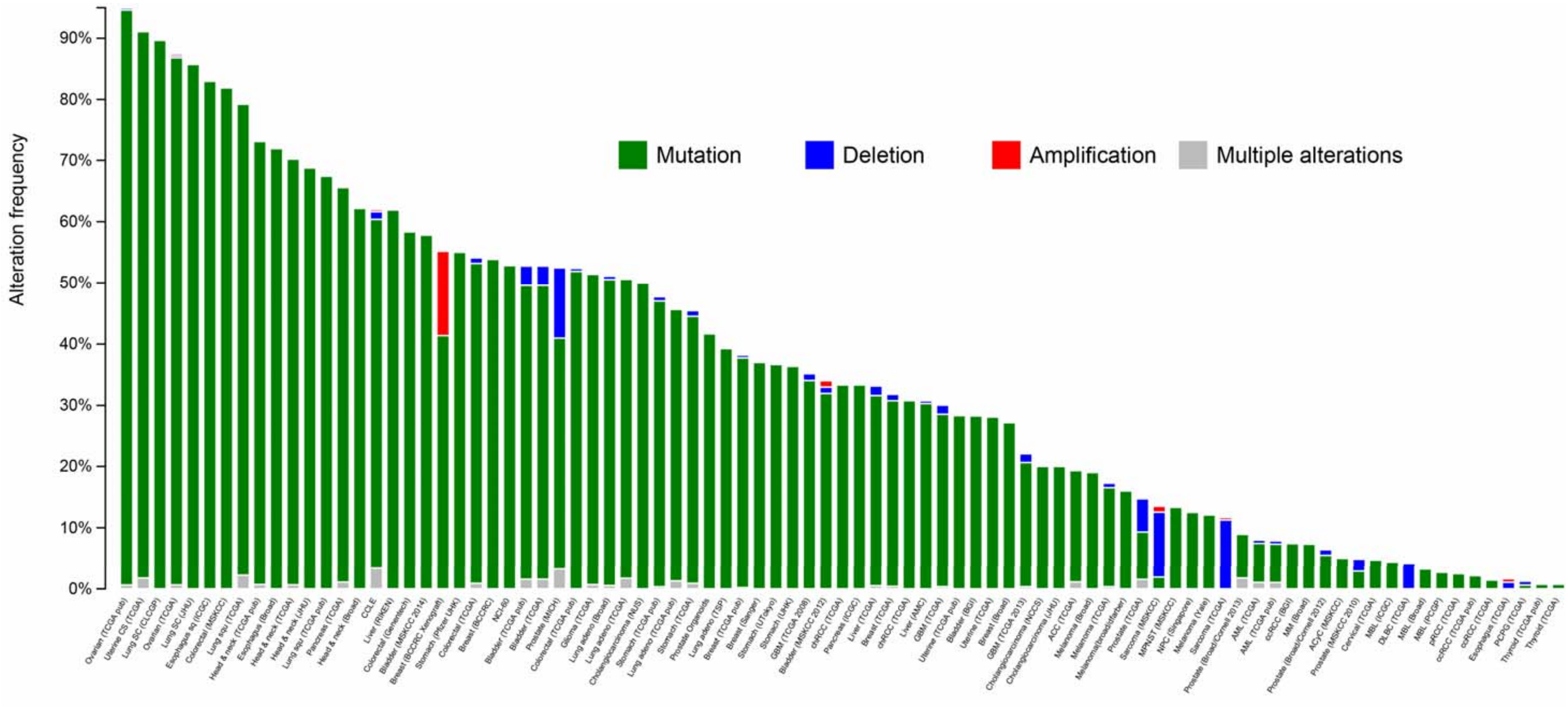

Figure S4. The genes targeted by the four miRNAs with copy number gain.

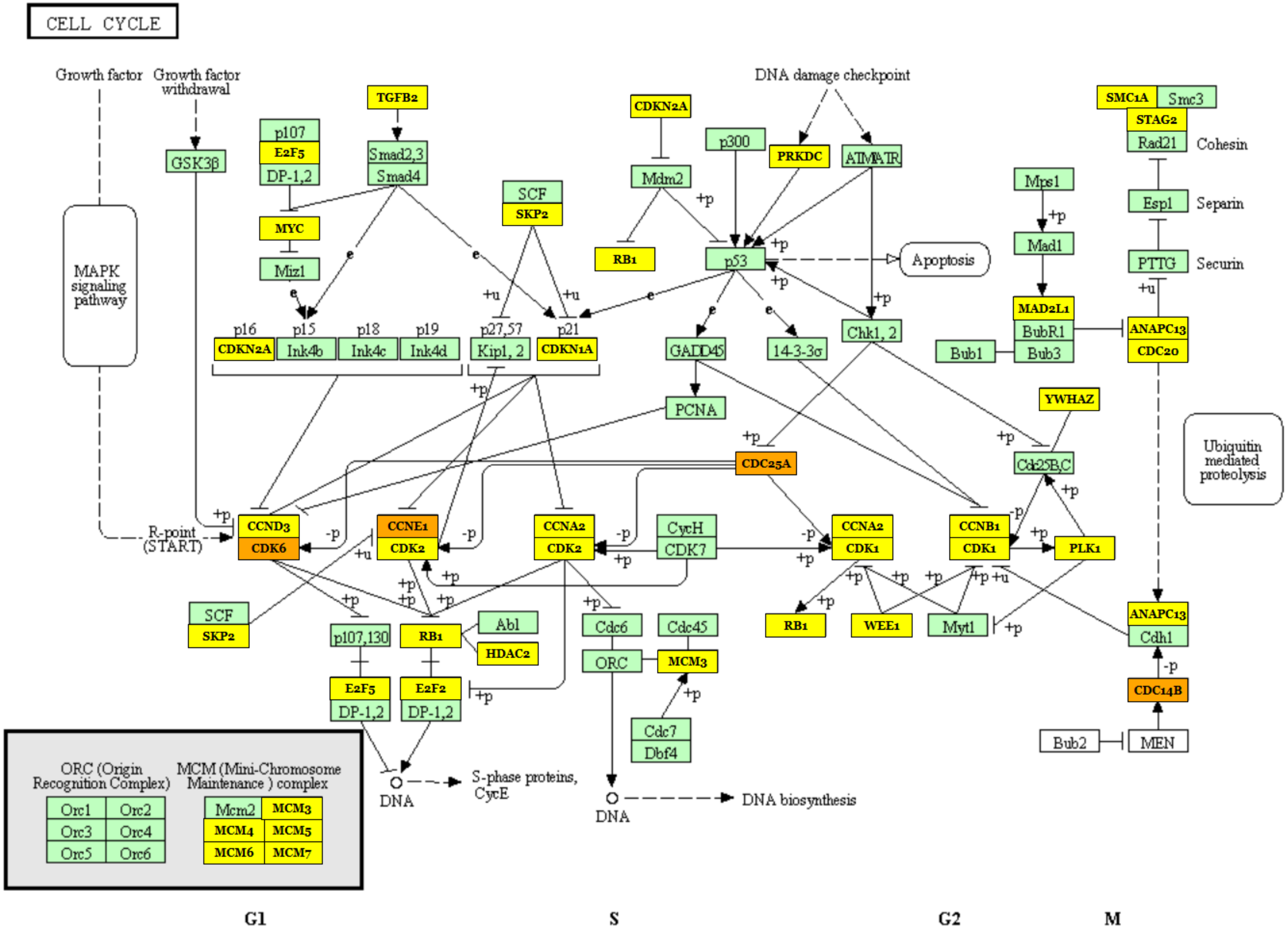

Figure S5. The mutational frequency of CRC-related genes with copy number variations in the TCGA CRC cohort.

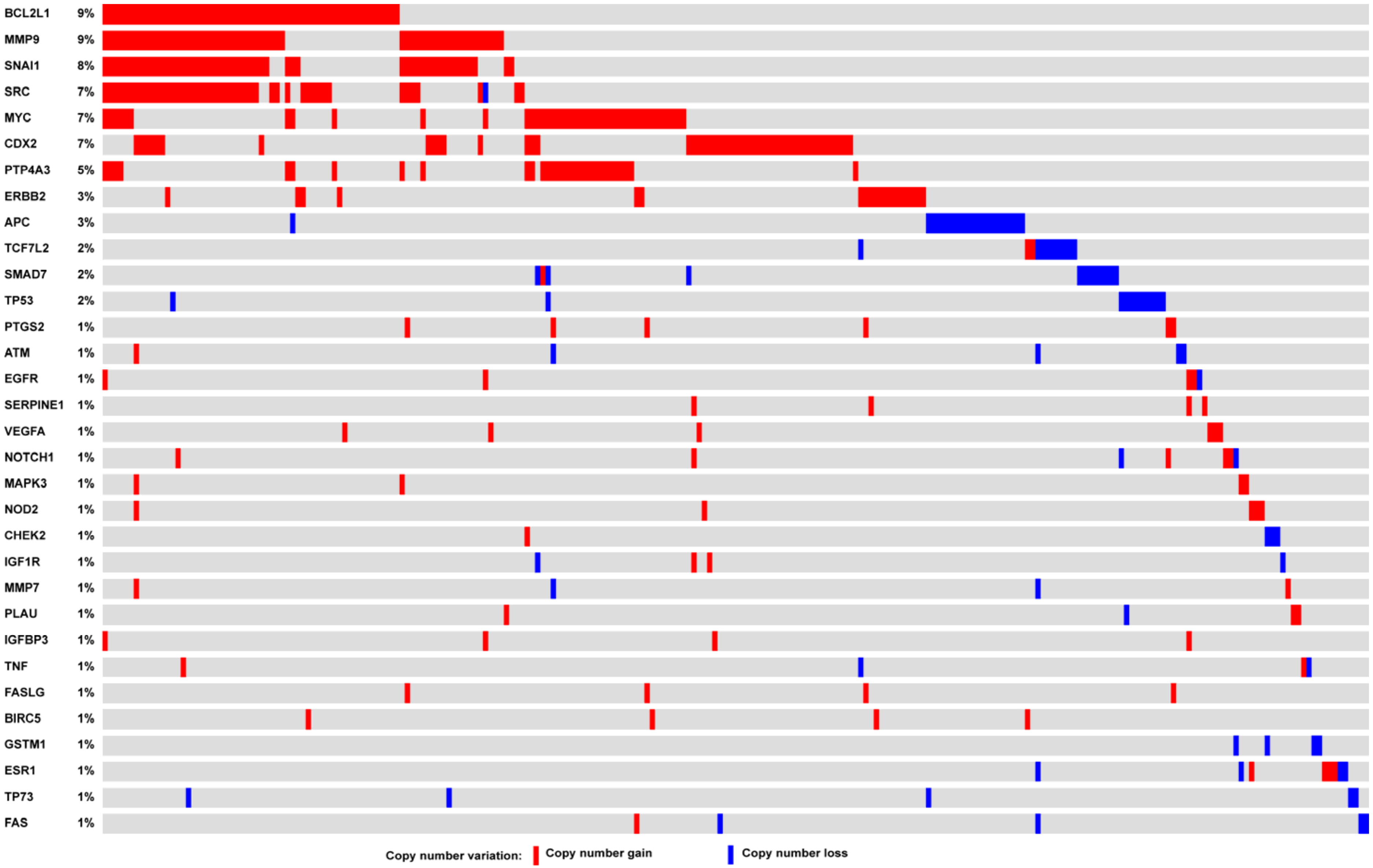

Figure S6. The concordance of copy number gain and gene up-regulation of BCL2L1.

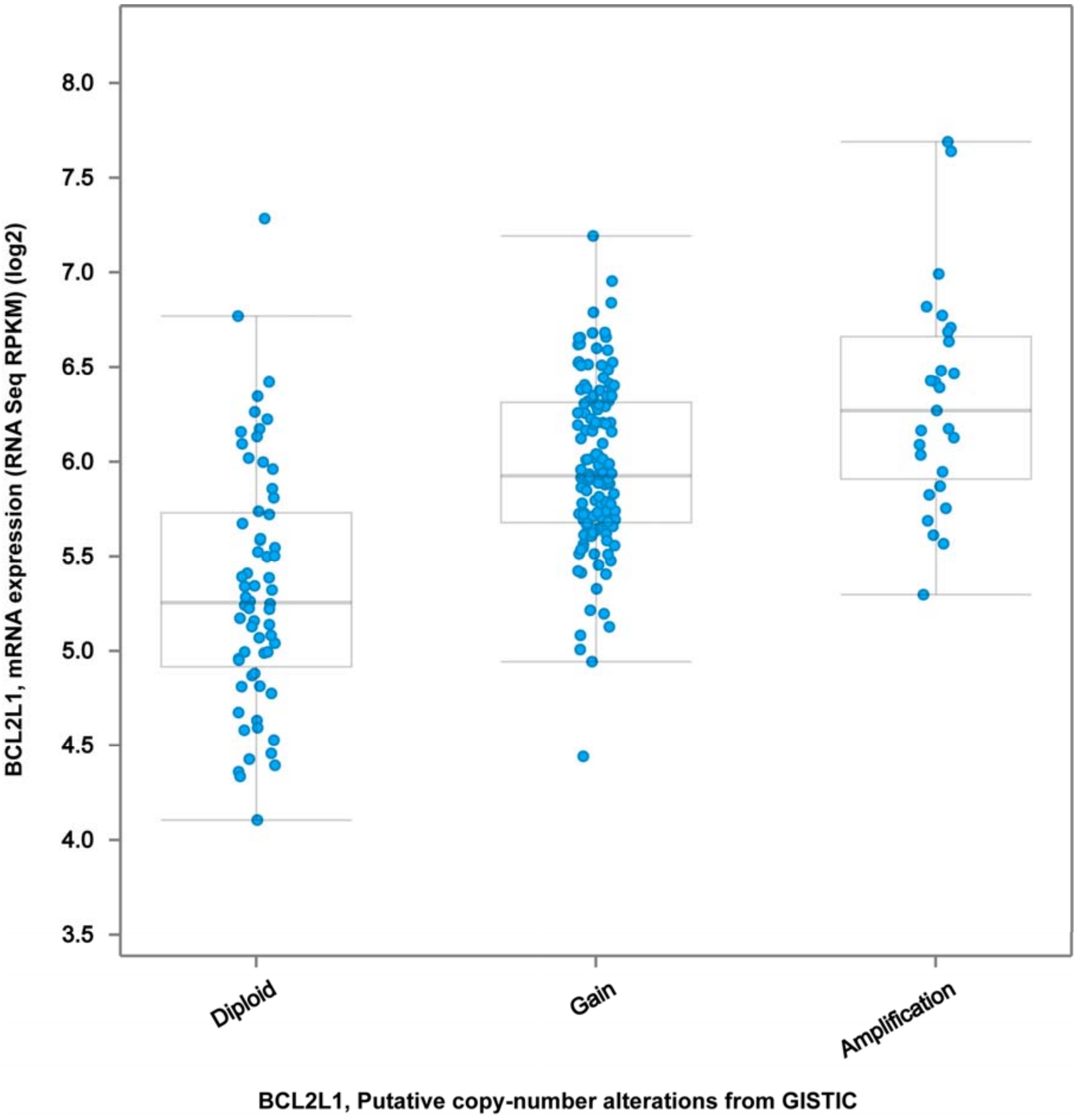

Supplement: Supplementary Dataset 1 [file srep30624-s1.zip › supplementary_data.pdf]
